# 7′-[4-(Trifluoro­meth­yl)phen­yl]-5′,6′,7′,7a’-tetra­hydrodispiro­[indan-2,5′-pyrrolo­[1,2-*c*][1,3]thia­zole-6′,2′′-indan]-1,3,1′′-trione

**DOI:** 10.1107/S1600536811047118

**Published:** 2011-11-12

**Authors:** Ang Chee Wei, Mohamed Ashraf Ali, Rusli Ismail, Ching Kheng Quah, Hoong-Kun Fun

**Affiliations:** a Institute for Research in Molecular Medicine, Universiti Sains Malaysia, 11800 USM, Penang, Malaysia; b X-ray Crystallography Unit, School of Physics, Universiti Sains Malaysia, 11800 USM, Penang, Malaysia

## Abstract

In the title compound, C_29_H_20_F_3_NO_3_S, the thia­zolidine ringadopts a half-chair conformation. The pyrrolidine and two five-membered carbocyclic rings are in envelope conformations with the spiro C atoms at the flaps. The trifluoro­methyl-substituted benzene ring forms dihedral angles of 62.37 (14) and 87.40 (14)° with the benzene rings of the dihydro-1*H*-indene units. The two benzene rings form a dihedral angle of 36.94 (15)°. The mol­ecular structure is stabilized by intra­molecular C—H⋯O hydrogen bonds, which generate *S*(6) ring motifs. In the crystal, mol­ecules are linked into inversion dimers by pairs of inter­molecular C—H⋯O hydrogen bonds, generating *R*_2_^2^(10) ring motifs.

## Related literature

For related structures, see: Wei, Ali, Choon *et al.* (2011*a* ▶,*b* ▶); Wei, Ali, Ismail *et al.* (2011 ▶); Wei, Ali, Yoon *et al.* (2011 ▶); Kumar *et al.* (2010 ▶). For hydrogen-bond motifs, see: Bernstein *et al.* (1995 ▶). For ring conformations, see: Cremer & Pople (1975 ▶). For bond-length data, see: Allen *et al.* (1987 ▶).
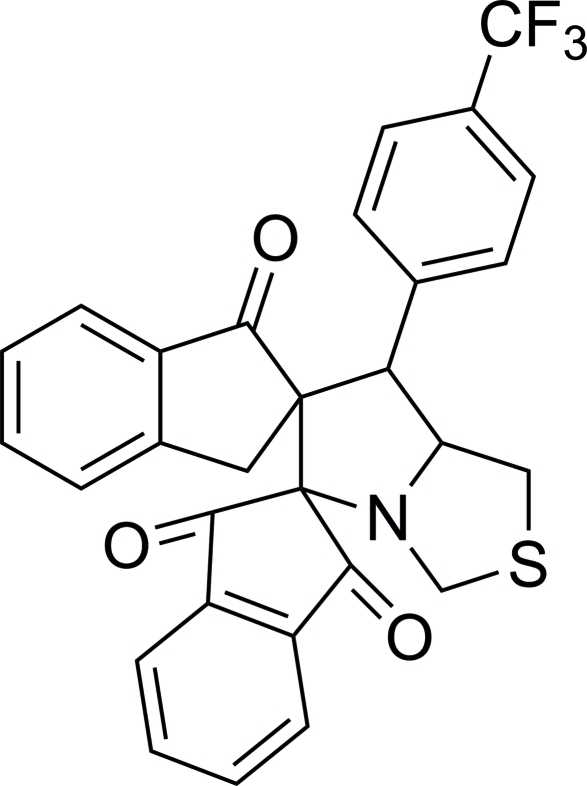


## Experimental

### 

#### Crystal data

C_29_H_20_F_3_NO_3_S
*M* *_r_* = 519.52
Triclinic, 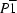

*a* = 8.5894 (9) Å
*b* = 12.3144 (13) Å
*c* = 12.6109 (15) Å
α = 68.181 (1)°
β = 87.589 (2)°
γ = 74.960 (2)°
*V* = 1193.7 (2) Å^3^
*Z* = 2
Mo *K*α radiation
μ = 0.19 mm^−1^
*T* = 296 K
0.23 × 0.22 × 0.09 mm

#### Data collection

Bruker SMART APEXII DUO CCD area-detector diffractometer
Absorption correction: multi-scan (*SADABS*; Bruker, 2009 ▶) *T*_min_ = 0.957, *T*_max_ = 0.983
25475 measured reflections
6922 independent reflections
4508 reflections with *I* > 2σ(*I*)
*R*_int_ = 0.038

#### Refinement

*R*[*F* ^2^ > 2σ(*F* ^2^)] = 0.063
*wR*(*F* ^2^) = 0.216
*S* = 1.05
6922 reflections
334 parameters
H-atom parameters constrained
Δρ_max_ = 0.73 e Å^−3^
Δρ_min_ = −0.69 e Å^−3^

### 

Data collection: *APEX2* (Bruker, 2009 ▶); cell refinement: *SAINT* (Bruker, 2009 ▶); data reduction: *SAINT*; program(s) used to solve structure: *SHELXTL* (Sheldrick, 2008 ▶); program(s) used to refine structure: *SHELXTL*; molecular graphics: *SHELXTL*; software used to prepare material for publication: *SHELXTL* and *PLATON* (Spek, 2009 ▶).

## Supplementary Material

Crystal structure: contains datablock(s) global, I. DOI: 10.1107/S1600536811047118/is5003sup1.cif

Structure factors: contains datablock(s) I. DOI: 10.1107/S1600536811047118/is5003Isup2.hkl

Additional supplementary materials:  crystallographic information; 3D view; checkCIF report

## Figures and Tables

**Table 1:** Hydrogen-bond geometry (Å, °)

*D*—H⋯*A*	*D*—H	H⋯*A*	*D*⋯*A*	*D*—H⋯*A*
C3—H3*A*⋯O1^i^	0.93	2.57	3.227 (3)	128
C18—H18*A*⋯O2	0.97	2.47	3.091 (3)	122
C19—H19*A*⋯O1	0.98	2.50	3.145 (3)	123

Symmetry code: (i) 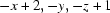.

**Fig. 1.**
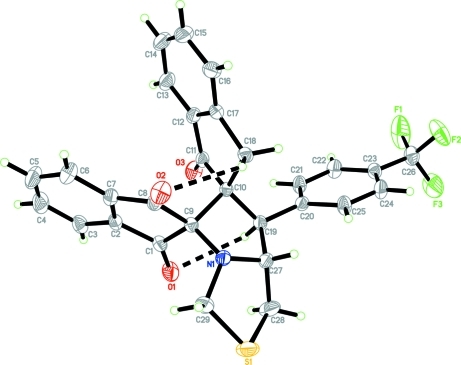
The molecular structure of the title compound, showing 30% probability displacement ellipsoids for non-H atoms. Intramolecular hydrogen bonds are shown as dashed lines.

**Fig. 2.**
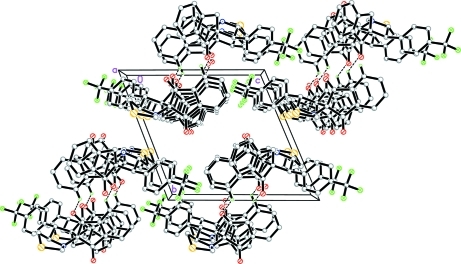
The crystal structure of the title compound, viewed along the a axis. H atoms not involved in hydrogen bonds (dashed lines) have been omitted for clarity.

## supplementary crystallographic information

### Comment

As part of our ongoing search for novel heterocyclic compounds with antitubercular activity (Wei, Ali, Choon *et al.*, 2011*a*,*b*; Wei, Ali, Ismail *et al.*, 2011; Wei, Ali, Yoon *et al.*, 2011), our group has synthesized the title compound as described below.

The molecular structure is shown in Fig. 1. The thiazolidine (S1/N1/C27-C29) ring is twisted about the S1—C28 bond, with puckering parameters (Cremer & Pople, 1975) Q = 0.405 (3) Å and φ = 204.5 (4)°, thereby adopting a half-chair conformation. The pyrrolidine (N1/C9/C10/C19/C27) and two five-membered carbocyclic (C1/C2/C7-C9 and C10-C12/C17/C18) rings are in envelope conformations, puckering parameters (Cremer & Pople, 1975) Q = 0.458 (2) Å and φ = 65.6 (3)° with atom C10 at the flap; Q = 0.178 (3) Å and φ = 322.2 (8)° with atom C9 at the flap; and Q = 0.202 (3) Å and φ = 352.7 (8)° with atom C10 at the flap, respectively. Bond lengths (Allen *et al.*, 1987) and angles are within normal ranges and are comparable to related structures (Kumar *et al.*, 2010; Wei, Ali, Choon *et al.*, 2011*a*,*b*; Wei, Ali, Ismail *et al.*, 2011; Wei, Ali, Yoon *et al.*, 2011). The trifluoromethyl-bound benzene (C20–C25) ring forms dihedral angles of 62.37 (14) and 87.40 (14)° with the other two benzene (C2–C7 and C12–C17) rings, respectively. The two benzene rings form a dihedral angle of 36.94 (15)°. The molecular structure is stabilized by intramolecular C18—H18A···O2 and C19—H19A···O1 hydrogen bonds (Table 1), which generate *S*(6) ring motifs (Fig. 1; Bernstein *et al.*, 1995).

In the crystal (Fig. 2), molecules are linked into inversion dimers by pairs of intermolecular C3—H3A···O1 hydrogen bonds (Table 1), generating ten-membered R^2^_2_(10) ring motifs (Bernstein *et al.*, 1995).

### Experimental

A mixture of (*Z*)-2-(4-trifluoromethylbenzylidene)-2,3-dihydro-1 *H*-indene-1-one (0.001 mmol), ninhydrin (0.001 mmol) and thiazolidine -4-carboxylic acid (0.002 mmol) (1:1:2) were dissolved in methanol (10 ml) and refluxed for 4 h. After completion of the reaction as evident from TLC, the mixture was poured into crushed ice. The precipitated solid was filtered, washed with water and recrystallised from petroleum ether-ethyl acetate mixture (1:1) to reveal the title compound as yellow crystals.

### Refinement

All H atoms were positioned geometrically and refined using a riding model with C—H = 0.93–0.98 Å and *U*_iso_(H) = 1.2*U*_eq_(C).

### Crystal data

**Table:**

C_29_H_20_F_3_NO_3_S	*Z* = 2
*M**_r_* = 519.52	*F*(000) = 536
Triclinic, *P*1	*D*_x_ = 1.445 Mg m^−^^3^
Hall symbol: -P 1	Mo *K*α radiation, λ = 0.71073 Å
*a* = 8.5894 (9) Å	Cell parameters from 5253 reflections
*b* = 12.3144 (13) Å	θ = 2.9–28.1°
*c* = 12.6109 (15) Å	µ = 0.19 mm^−^^1^
α = 68.181 (1)°	*T* = 296 K
β = 87.589 (2)°	Block, yellow
γ = 74.960 (2)°	0.23 × 0.22 × 0.09 mm
*V* = 1193.7 (2) Å^3^	

### Data collection

**Table:**

Bruker SMART APEXII DUO CCD area-detector diffractometer	6922 independent reflections
Radiation source: fine-focus sealed tube	4508 reflections with *I* > 2σ(*I*)
graphite	*R*_int_ = 0.038
φ and ω scans	θ_max_ = 30.1°, θ_min_ = 1.7°
Absorption correction: multi-scan (*SADABS*; Bruker, 2009)	*h* = −12→12
*T*_min_ = 0.957, *T*_max_ = 0.983	*k* = −17→17
25475 measured reflections	*l* = −17→17

### Refinement

**Table:**

Refinement on *F*^2^	Primary atom site location: structure-invariant direct methods
Least-squares matrix: full	Secondary atom site location: difference Fourier map
*R*[*F*^2^ > 2σ(*F*^2^)] = 0.063	Hydrogen site location: inferred from neighbouring sites
*wR*(*F*^2^) = 0.216	H-atom parameters constrained
*S* = 1.05	*w* = 1/[σ^2^(*F*_o_^2^) + (0.1159*P*)^2^ + 0.3319*P*] where *P* = (*F*_o_^2^ + 2*F*_c_^2^)/3
6922 reflections	(Δ/σ)_max_ = 0.001
334 parameters	Δρ_max_ = 0.73 e Å^−^^3^
0 restraints	Δρ_min_ = −0.69 e Å^−^^3^

### Special details

**Table:**

Geometry. All esds (except the esd in the dihedral angle between two l.s. planes) are estimated using the full covariance matrix. The cell esds are taken into account individually in the estimation of esds in distances, angles and torsion angles; correlations between esds in cell parameters are only used when they are defined by crystal symmetry. An approximate (isotropic) treatment of cell esds is used for estimating esds involving l.s. planes.
Refinement. Refinement of F^2^ against ALL reflections. The weighted R-factor wR and goodness of fit S are based on F^2^, conventional R-factors R are based on F, with F set to zero for negative F^2^. The threshold expression of F^2^ > 2sigma(F^2^) is used only for calculating R-factors(gt) etc. and is not relevant to the choice of reflections for refinement. R-factors based on F^2^ are statistically about twice as large as those based on F, and R- factors based on ALL data will be even larger.

### Fractional atomic coordinates and isotropic or equivalent isotropic displacement parameters (Å^2^)

**Table:**

	*x*	*y*	*z*	*U*_iso_*/*U*_eq_	
S1	0.96436 (9)	0.35285 (8)	0.03916 (7)	0.0605 (2)	
F1	0.0800 (4)	0.0579 (3)	−0.1089 (2)	0.1228 (12)	
F2	0.0364 (3)	0.23905 (19)	−0.2219 (2)	0.0861 (7)	
F3	0.2346 (3)	0.1062 (3)	−0.2398 (2)	0.1037 (9)	
O1	0.8556 (2)	0.10792 (17)	0.32038 (17)	0.0543 (5)	
O2	0.5498 (3)	0.48429 (15)	0.31011 (16)	0.0504 (5)	
O3	0.4883 (2)	0.07066 (15)	0.36158 (15)	0.0499 (4)	
N1	0.7039 (2)	0.36282 (17)	0.15914 (15)	0.0335 (4)	
C1	0.7605 (3)	0.1814 (2)	0.34956 (19)	0.0360 (5)	
C2	0.7386 (3)	0.1793 (2)	0.46658 (19)	0.0371 (5)	
C3	0.8044 (3)	0.0862 (2)	0.5701 (2)	0.0469 (6)	
H3A	0.8676	0.0115	0.5723	0.056*	
C4	0.7723 (4)	0.1090 (3)	0.6684 (2)	0.0622 (8)	
H4A	0.8148	0.0483	0.7383	0.075*	
C5	0.6787 (5)	0.2192 (3)	0.6666 (2)	0.0678 (9)	
H5A	0.6596	0.2312	0.7351	0.081*	
C6	0.6127 (4)	0.3124 (3)	0.5648 (2)	0.0565 (7)	
H6A	0.5489	0.3865	0.5638	0.068*	
C7	0.6452 (3)	0.2912 (2)	0.46404 (19)	0.0384 (5)	
C8	0.6005 (3)	0.3756 (2)	0.34347 (19)	0.0361 (5)	
C9	0.6437 (2)	0.29994 (18)	0.26748 (17)	0.0303 (4)	
C10	0.4929 (2)	0.27225 (18)	0.22533 (16)	0.0297 (4)	
C11	0.4296 (3)	0.1790 (2)	0.32498 (17)	0.0346 (4)	
C12	0.2880 (3)	0.2481 (2)	0.36452 (19)	0.0386 (5)	
C13	0.2053 (4)	0.2083 (3)	0.4635 (2)	0.0522 (7)	
H13A	0.2355	0.1281	0.5145	0.063*	
C14	0.0782 (4)	0.2912 (3)	0.4831 (3)	0.0622 (8)	
H14A	0.0208	0.2669	0.5482	0.075*	
C15	0.0340 (4)	0.4113 (3)	0.4068 (3)	0.0624 (8)	
H15A	−0.0526	0.4660	0.4218	0.075*	
C16	0.1164 (3)	0.4511 (3)	0.3090 (2)	0.0496 (6)	
H16A	0.0868	0.5316	0.2588	0.060*	
C17	0.2450 (3)	0.3672 (2)	0.28753 (19)	0.0362 (5)	
C18	0.3494 (3)	0.3862 (2)	0.18652 (18)	0.0352 (4)	
H18A	0.3856	0.4589	0.1694	0.042*	
H18B	0.2912	0.3927	0.1192	0.042*	
C19	0.5698 (3)	0.22744 (19)	0.13116 (17)	0.0319 (4)	
H19A	0.6447	0.1477	0.1703	0.038*	
C20	0.4566 (3)	0.21104 (19)	0.05324 (17)	0.0329 (4)	
C21	0.4027 (3)	0.1066 (2)	0.09141 (19)	0.0422 (5)	
H21A	0.4346	0.0500	0.1651	0.051*	
C22	0.3029 (4)	0.0844 (2)	0.0232 (2)	0.0468 (6)	
H22A	0.2671	0.0144	0.0513	0.056*	
C23	0.2565 (3)	0.1670 (2)	−0.08730 (19)	0.0388 (5)	
C24	0.3089 (3)	0.2709 (2)	−0.1275 (2)	0.0464 (6)	
H24A	0.2788	0.3262	−0.2019	0.056*	
C25	0.4062 (3)	0.2935 (2)	−0.0577 (2)	0.0438 (6)	
H25A	0.4385	0.3650	−0.0853	0.053*	
C26	0.1537 (4)	0.1431 (2)	−0.1638 (2)	0.0507 (6)	
C27	0.6737 (3)	0.3150 (2)	0.07265 (18)	0.0363 (5)	
H27A	0.6121	0.3817	0.0059	0.044*	
C28	0.8365 (4)	0.2580 (3)	0.0364 (3)	0.0570 (7)	
H28A	0.8261	0.2578	−0.0398	0.068*	
H28B	0.8801	0.1753	0.0894	0.068*	
C29	0.8651 (3)	0.3807 (3)	0.1593 (2)	0.0479 (6)	
H29A	0.9261	0.3252	0.2302	0.058*	
H29B	0.8578	0.4629	0.1526	0.058*	

### Atomic displacement parameters (Å^2^)

**Table:**

	*U*^11^	*U*^22^	*U*^33^	*U*^12^	*U*^13^	*U*^23^
S1	0.0445 (4)	0.0840 (6)	0.0729 (5)	−0.0327 (4)	0.0244 (3)	−0.0430 (4)
F1	0.182 (3)	0.124 (2)	0.0866 (16)	−0.110 (2)	−0.0369 (17)	−0.0150 (14)
F2	0.0781 (14)	0.0770 (13)	0.1073 (17)	0.0055 (11)	−0.0465 (13)	−0.0507 (12)
F3	0.0924 (16)	0.151 (2)	0.0966 (17)	0.0001 (15)	−0.0160 (13)	−0.0971 (17)
O1	0.0494 (10)	0.0514 (11)	0.0527 (11)	0.0130 (9)	−0.0126 (9)	−0.0248 (9)
O2	0.0688 (12)	0.0320 (9)	0.0500 (10)	−0.0087 (8)	0.0040 (9)	−0.0178 (7)
O3	0.0666 (12)	0.0343 (9)	0.0400 (9)	−0.0134 (8)	0.0011 (8)	−0.0038 (7)
N1	0.0341 (9)	0.0377 (9)	0.0316 (8)	−0.0148 (8)	0.0040 (7)	−0.0127 (7)
C1	0.0337 (11)	0.0358 (11)	0.0367 (11)	−0.0051 (9)	−0.0052 (9)	−0.0134 (9)
C2	0.0371 (11)	0.0405 (12)	0.0341 (10)	−0.0127 (9)	−0.0038 (9)	−0.0120 (9)
C3	0.0472 (14)	0.0457 (13)	0.0394 (12)	−0.0132 (11)	−0.0091 (10)	−0.0047 (10)
C4	0.072 (2)	0.071 (2)	0.0339 (13)	−0.0281 (17)	−0.0066 (13)	−0.0021 (13)
C5	0.085 (2)	0.090 (2)	0.0337 (13)	−0.030 (2)	0.0109 (14)	−0.0248 (14)
C6	0.0712 (19)	0.0616 (17)	0.0434 (14)	−0.0172 (15)	0.0099 (13)	−0.0280 (13)
C7	0.0431 (12)	0.0413 (12)	0.0334 (10)	−0.0130 (10)	0.0018 (9)	−0.0156 (9)
C8	0.0391 (11)	0.0354 (11)	0.0350 (11)	−0.0090 (9)	0.0023 (9)	−0.0149 (9)
C9	0.0304 (10)	0.0296 (9)	0.0296 (9)	−0.0064 (8)	−0.0007 (7)	−0.0102 (8)
C10	0.0300 (9)	0.0288 (9)	0.0260 (9)	−0.0065 (8)	−0.0003 (7)	−0.0062 (7)
C11	0.0410 (11)	0.0351 (11)	0.0254 (9)	−0.0144 (9)	−0.0002 (8)	−0.0056 (8)
C12	0.0415 (12)	0.0496 (13)	0.0311 (10)	−0.0207 (10)	0.0053 (9)	−0.0166 (9)
C13	0.0595 (16)	0.0697 (18)	0.0379 (12)	−0.0341 (15)	0.0137 (11)	−0.0216 (12)
C14	0.0572 (17)	0.102 (3)	0.0465 (15)	−0.0369 (18)	0.0211 (13)	−0.0405 (17)
C15	0.0451 (15)	0.097 (2)	0.0654 (19)	−0.0165 (16)	0.0124 (13)	−0.0555 (19)
C16	0.0370 (12)	0.0615 (16)	0.0567 (15)	−0.0074 (11)	0.0008 (11)	−0.0327 (13)
C17	0.0303 (10)	0.0467 (12)	0.0350 (10)	−0.0114 (9)	0.0004 (8)	−0.0180 (9)
C18	0.0312 (10)	0.0339 (10)	0.0335 (10)	−0.0046 (8)	−0.0018 (8)	−0.0070 (8)
C19	0.0330 (10)	0.0330 (10)	0.0289 (9)	−0.0097 (8)	0.0006 (8)	−0.0101 (8)
C20	0.0352 (10)	0.0347 (10)	0.0293 (9)	−0.0115 (9)	0.0023 (8)	−0.0108 (8)
C21	0.0542 (14)	0.0390 (12)	0.0321 (10)	−0.0201 (11)	−0.0032 (10)	−0.0059 (9)
C22	0.0642 (16)	0.0409 (13)	0.0417 (12)	−0.0262 (12)	−0.0007 (11)	−0.0137 (10)
C23	0.0413 (12)	0.0410 (12)	0.0376 (11)	−0.0089 (10)	−0.0019 (9)	−0.0194 (9)
C24	0.0577 (15)	0.0471 (13)	0.0304 (11)	−0.0175 (12)	−0.0057 (10)	−0.0067 (9)
C25	0.0559 (14)	0.0404 (12)	0.0336 (11)	−0.0233 (11)	−0.0052 (10)	−0.0042 (9)
C26	0.0620 (17)	0.0474 (14)	0.0468 (14)	−0.0112 (13)	−0.0080 (12)	−0.0232 (11)
C27	0.0381 (11)	0.0443 (12)	0.0313 (10)	−0.0175 (10)	0.0048 (8)	−0.0152 (9)
C28	0.0521 (15)	0.0745 (19)	0.0665 (18)	−0.0302 (15)	0.0277 (13)	−0.0445 (16)
C29	0.0423 (13)	0.0613 (16)	0.0507 (14)	−0.0265 (12)	0.0071 (11)	−0.0245 (12)

### Geometric parameters (Å, °)

**Table:**

S1—C29	1.807 (3)	C13—C14	1.372 (5)
S1—C28	1.809 (3)	C13—H13A	0.9300
F1—C26	1.322 (4)	C14—C15	1.393 (5)
F2—C26	1.323 (3)	C14—H14A	0.9300
F3—C26	1.308 (4)	C15—C16	1.386 (4)
O1—C1	1.209 (3)	C15—H15A	0.9300
O2—C8	1.205 (3)	C16—C17	1.396 (4)
O3—C11	1.208 (3)	C16—H16A	0.9300
N1—C9	1.447 (3)	C17—C18	1.508 (3)
N1—C29	1.458 (3)	C18—H18A	0.9700
N1—C27	1.478 (3)	C18—H18B	0.9700
C1—C2	1.471 (3)	C19—C20	1.510 (3)
C1—C9	1.553 (3)	C19—C27	1.534 (3)
C2—C7	1.393 (3)	C19—H19A	0.9800
C2—C3	1.397 (3)	C20—C21	1.389 (3)
C3—C4	1.371 (4)	C20—C25	1.394 (3)
C3—H3A	0.9300	C21—C22	1.382 (3)
C4—C5	1.379 (5)	C21—H21A	0.9300
C4—H4A	0.9300	C22—C23	1.384 (3)
C5—C6	1.383 (4)	C22—H22A	0.9300
C5—H5A	0.9300	C23—C24	1.377 (3)
C6—C7	1.393 (3)	C23—C26	1.488 (3)
C6—H6A	0.9300	C24—C25	1.384 (3)
C7—C8	1.485 (3)	C24—H24A	0.9300
C8—C9	1.542 (3)	C25—H25A	0.9300
C9—C10	1.581 (3)	C27—C28	1.528 (4)
C10—C18	1.540 (3)	C27—H27A	0.9800
C10—C19	1.544 (3)	C28—H28A	0.9700
C10—C11	1.545 (3)	C28—H28B	0.9700
C11—C12	1.476 (3)	C29—H29A	0.9700
C12—C17	1.386 (3)	C29—H29B	0.9700
C12—C13	1.398 (3)		
			
C29—S1—C28	91.51 (12)	C12—C17—C16	119.6 (2)
C9—N1—C29	118.30 (18)	C12—C17—C18	111.7 (2)
C9—N1—C27	110.40 (16)	C16—C17—C18	128.7 (2)
C29—N1—C27	113.82 (18)	C17—C18—C10	104.01 (17)
O1—C1—C2	127.1 (2)	C17—C18—H18A	111.0
O1—C1—C9	125.3 (2)	C10—C18—H18A	111.0
C2—C1—C9	107.46 (18)	C17—C18—H18B	111.0
C7—C2—C3	120.8 (2)	C10—C18—H18B	111.0
C7—C2—C1	110.33 (19)	H18A—C18—H18B	109.0
C3—C2—C1	128.8 (2)	C20—C19—C27	116.21 (17)
C4—C3—C2	117.6 (3)	C20—C19—C10	116.84 (18)
C4—C3—H3A	121.2	C27—C19—C10	103.12 (16)
C2—C3—H3A	121.2	C20—C19—H19A	106.6
C3—C4—C5	121.9 (3)	C27—C19—H19A	106.6
C3—C4—H4A	119.0	C10—C19—H19A	106.6
C5—C4—H4A	119.0	C21—C20—C25	117.3 (2)
C4—C5—C6	121.2 (3)	C21—C20—C19	119.05 (18)
C4—C5—H5A	119.4	C25—C20—C19	123.65 (19)
C6—C5—H5A	119.4	C22—C21—C20	121.9 (2)
C5—C6—C7	117.6 (3)	C22—C21—H21A	119.0
C5—C6—H6A	121.2	C20—C21—H21A	119.0
C7—C6—H6A	121.2	C21—C22—C23	119.6 (2)
C6—C7—C2	120.8 (2)	C21—C22—H22A	120.2
C6—C7—C8	129.5 (2)	C23—C22—H22A	120.2
C2—C7—C8	109.64 (19)	C24—C23—C22	119.6 (2)
O2—C8—C7	126.4 (2)	C24—C23—C26	119.7 (2)
O2—C8—C9	126.0 (2)	C22—C23—C26	120.6 (2)
C7—C8—C9	107.47 (18)	C23—C24—C25	120.3 (2)
N1—C9—C8	113.35 (17)	C23—C24—H24A	119.9
N1—C9—C1	116.89 (18)	C25—C24—H24A	119.9
C8—C9—C1	101.91 (16)	C24—C25—C20	121.2 (2)
N1—C9—C10	100.82 (15)	C24—C25—H25A	119.4
C8—C9—C10	113.59 (17)	C20—C25—H25A	119.4
C1—C9—C10	110.80 (16)	F3—C26—F1	104.7 (3)
C18—C10—C19	116.52 (16)	F3—C26—F2	106.1 (2)
C18—C10—C11	104.02 (17)	F1—C26—F2	104.9 (3)
C19—C10—C11	115.45 (17)	F3—C26—C23	113.3 (3)
C18—C10—C9	110.58 (16)	F1—C26—C23	113.7 (2)
C19—C10—C9	99.45 (16)	F2—C26—C23	113.3 (2)
C11—C10—C9	110.96 (16)	N1—C27—C28	108.28 (19)
O3—C11—C12	128.1 (2)	N1—C27—C19	104.79 (16)
O3—C11—C10	125.1 (2)	C28—C27—C19	115.4 (2)
C12—C11—C10	106.86 (18)	N1—C27—H27A	109.4
C17—C12—C13	121.9 (3)	C28—C27—H27A	109.4
C17—C12—C11	109.28 (19)	C19—C27—H27A	109.4
C13—C12—C11	128.8 (2)	C27—C28—S1	104.62 (18)
C14—C13—C12	117.9 (3)	C27—C28—H28A	110.8
C14—C13—H13A	121.0	S1—C28—H28A	110.8
C12—C13—H13A	121.0	C27—C28—H28B	110.8
C13—C14—C15	120.8 (3)	S1—C28—H28B	110.8
C13—C14—H14A	119.6	H28A—C28—H28B	108.9
C15—C14—H14A	119.6	N1—C29—S1	108.05 (16)
C16—C15—C14	121.4 (3)	N1—C29—H29A	110.1
C16—C15—H15A	119.3	S1—C29—H29A	110.1
C14—C15—H15A	119.3	N1—C29—H29B	110.1
C15—C16—C17	118.4 (3)	S1—C29—H29B	110.1
C15—C16—H16A	120.8	H29A—C29—H29B	108.4
C17—C16—H16A	120.8		
			
O1—C1—C2—C7	166.6 (2)	C17—C12—C13—C14	0.0 (4)
C9—C1—C2—C7	−10.8 (2)	C11—C12—C13—C14	178.4 (2)
O1—C1—C2—C3	−9.7 (4)	C12—C13—C14—C15	−0.3 (4)
C9—C1—C2—C3	173.0 (2)	C13—C14—C15—C16	0.0 (4)
C7—C2—C3—C4	0.6 (4)	C14—C15—C16—C17	0.6 (4)
C1—C2—C3—C4	176.5 (2)	C13—C12—C17—C16	0.6 (3)
C2—C3—C4—C5	0.0 (4)	C11—C12—C17—C16	−178.1 (2)
C3—C4—C5—C6	0.0 (5)	C13—C12—C17—C18	−178.3 (2)
C4—C5—C6—C7	−0.5 (5)	C11—C12—C17—C18	3.0 (2)
C5—C6—C7—C2	1.1 (4)	C15—C16—C17—C12	−0.9 (3)
C5—C6—C7—C8	−175.6 (3)	C15—C16—C17—C18	177.8 (2)
C3—C2—C7—C6	−1.2 (4)	C12—C17—C18—C10	−14.8 (2)
C1—C2—C7—C6	−177.8 (2)	C16—C17—C18—C10	166.4 (2)
C3—C2—C7—C8	176.1 (2)	C19—C10—C18—C17	148.04 (17)
C1—C2—C7—C8	−0.5 (3)	C11—C10—C18—C17	19.8 (2)
C6—C7—C8—O2	13.2 (4)	C9—C10—C18—C17	−99.42 (18)
C2—C7—C8—O2	−163.8 (2)	C18—C10—C19—C20	−50.9 (2)
C6—C7—C8—C9	−171.4 (3)	C11—C10—C19—C20	71.6 (2)
C2—C7—C8—C9	11.7 (2)	C9—C10—C19—C20	−169.66 (17)
C29—N1—C9—C8	72.0 (3)	C18—C10—C19—C27	77.9 (2)
C27—N1—C9—C8	−154.43 (18)	C11—C10—C19—C27	−159.62 (17)
C29—N1—C9—C1	−46.1 (3)	C9—C10—C19—C27	−40.89 (18)
C27—N1—C9—C1	87.5 (2)	C27—C19—C20—C21	157.7 (2)
C29—N1—C9—C10	−166.28 (19)	C10—C19—C20—C21	−80.1 (3)
C27—N1—C9—C10	−32.7 (2)	C27—C19—C20—C25	−20.1 (3)
O2—C8—C9—N1	32.0 (3)	C10—C19—C20—C25	102.1 (3)
C7—C8—C9—N1	−143.50 (18)	C25—C20—C21—C22	−0.1 (4)
O2—C8—C9—C1	158.5 (2)	C19—C20—C21—C22	−178.0 (2)
C7—C8—C9—C1	−17.0 (2)	C20—C21—C22—C23	1.0 (4)
O2—C8—C9—C10	−82.3 (3)	C21—C22—C23—C24	−0.7 (4)
C7—C8—C9—C10	102.2 (2)	C21—C22—C23—C26	177.9 (3)
O1—C1—C9—N1	−36.5 (3)	C22—C23—C24—C25	−0.6 (4)
C2—C1—C9—N1	140.89 (18)	C26—C23—C24—C25	−179.2 (3)
O1—C1—C9—C8	−160.6 (2)	C23—C24—C25—C20	1.6 (4)
C2—C1—C9—C8	16.8 (2)	C21—C20—C25—C24	−1.2 (4)
O1—C1—C9—C10	78.2 (3)	C19—C20—C25—C24	176.6 (2)
C2—C1—C9—C10	−104.41 (19)	C24—C23—C26—F3	76.1 (3)
N1—C9—C10—C18	−78.39 (19)	C22—C23—C26—F3	−102.5 (3)
C8—C9—C10—C18	43.2 (2)	C24—C23—C26—F1	−164.5 (3)
C1—C9—C10—C18	157.20 (17)	C22—C23—C26—F1	16.9 (4)
N1—C9—C10—C19	44.70 (18)	C24—C23—C26—F2	−44.9 (4)
C8—C9—C10—C19	166.29 (16)	C22—C23—C26—F2	136.5 (3)
C1—C9—C10—C19	−79.71 (19)	C9—N1—C27—C28	−116.7 (2)
N1—C9—C10—C11	166.72 (17)	C29—N1—C27—C28	19.2 (3)
C8—C9—C10—C11	−71.7 (2)	C9—N1—C27—C19	7.0 (2)
C1—C9—C10—C11	42.3 (2)	C29—N1—C27—C19	142.8 (2)
C18—C10—C11—O3	161.5 (2)	C20—C19—C27—N1	151.74 (19)
C19—C10—C11—O3	32.5 (3)	C10—C19—C27—N1	22.6 (2)
C9—C10—C11—O3	−79.6 (3)	C20—C19—C27—C28	−89.3 (3)
C18—C10—C11—C12	−18.8 (2)	C10—C19—C27—C28	141.6 (2)
C19—C10—C11—C12	−147.68 (18)	N1—C27—C28—S1	−35.5 (2)
C9—C10—C11—C12	100.17 (19)	C19—C27—C28—S1	−152.48 (17)
O3—C11—C12—C17	−170.0 (2)	C29—S1—C28—C27	34.0 (2)
C10—C11—C12—C17	10.3 (2)	C9—N1—C29—S1	138.59 (17)
O3—C11—C12—C13	11.5 (4)	C27—N1—C29—S1	6.5 (3)
C10—C11—C12—C13	−168.3 (2)	C28—S1—C29—N1	−24.1 (2)

### Hydrogen-bond geometry (Å, °)

**Table:**

*D*—H···*A*	*D*—H	H···*A*	*D*···*A*	*D*—H···*A*
C3—H3A···O1^i^	0.93	2.57	3.227 (3)	128.
C18—H18A···O2	0.97	2.47	3.091 (3)	122.
C19—H19A···O1	0.98	2.50	3.145 (3)	123.

Symmetry codes: (i) −*x*+2, −*y*, −*z*+1.
